# First sliding Amyand hernia harbouring appendicular schistosomiasis: Case report

**DOI:** 10.1016/j.ijscr.2019.09.014

**Published:** 2019-09-20

**Authors:** Ali Toffaha, Walid El Ansari, Orwa Elaiwy, Munzir Obaid, Omer Al-Yahri, Sherif Abdelazim

**Affiliations:** a Department of General Surgery, Hamad Medical Corporation, Doha, Qatar; b Department of Surgery, Hamad General Hospital, Hamad Medical Corporation, Doha, Qatar; c College of Medicine, Qatar University, Doha, Qatar; d School of Health and Education, University of Skövde, Skövde, Sweden; e Department of Laboratory Medicine and Pathology, Hamad Medical Corporation, Doha, Qatar

**Keywords:** Inguinal hernia, Intestinal schistosomiasis, Tropical disease, Case report

## Abstract

**Introduction:**

Amyand’s hernia (AH) is rare, schistosomiasis of the appendix is very uncommon, and both conditions coexisting together is an extremely rare event. Pre-operative diagnosis of each of the two conditions is usually difficult. To the best of our knowledge, the current paper is first to report both these two conditions in coexistence.

**Presentation of case:**

A 31-year old man who had no comorbidities was admitted electively as a day case of non-complicated right indirect inguinal hernia. Further history and physical examination were unremarkable. Intraoperatively the patient was found to have right sliding AH with appendicular schistosomiasis (AS). The patient underwent Lichtenstein repair of the hernia with appendectomy. On follow up he was referred to infectious disease clinic, and the post-operative course was uneventful.

**Conclusions:**

Intraoperative identification of non-typical hernia sac before its opening should alert the surgeon to the possibility of sliding hernia and the presence of an organ as a part of the sac. Rare causes of appendicular masses like schistosomiasis granuloma should be considered in endemic areas or immigrants from these areas, despite the difficulty of preoperative diagnosis. Management should follow general guidelines of appendectomy, hernia repair and dealing with the associated pathology if present.

## Highlights

• Combination of Amyand hernia and appendicular schistosomiasis can happen.
• Appendicular schistosomiasis should be considered in immigrants from endemic areas.
• Atypical thick hernia sac intraoperatively showed be managed carefully.
• First reported case of Amyand hernia and appendicular schistosomiasis.

## 1 Introduction

Amyand’s hernia (AH), is the rare presence of appendix in the inguinal hernial sac [1], has an estimated incidence of 1% of all inguinal hernias [2]. The clinical picture of AH depends on whether the appendix progresses to inflammation (≈0.1% of appendicitis cases) [3], or is further incarcerated leading to strangulation and perforation [4]. AH occurs mostly in males [5], at all ages [6]. Moreover, schistosomiasis of the appendix is rare even in endemic areas [7]. It features submucosal and serosal granulomatous inflammation [8], initially well-demarcated epithelioid granulomas and tissue eosinophilia that may progress to fibrous replacement and hyalinization [8]. The current paper reports the first ever sliding Amyand hernia coexisting with appendicular schistosomiasis. The patient was adult male presenting with right inguinal hernia, diagnosis was intraoperative, mesh hernioplasty with appendectomy were performed, and the post-operative course was uneventful. We report this case in line with the updated consensus-based surgical case report (SCARE) guidelines [9].

## 2 Case presentation

A 31-year old Tanzanian man presented to the Surgery clinic at *** General Hospital (an academic hospital of *** Medical Corporation, Doha, Qatar) with two-year history of right groin swelling that becomes more prominent with standing and exertion, with episodes of discomfort in the same area but without intense pain. He had no comorbidities, there were no other gastrointestinal or urologic symptoms, and the systemic review was unremarkable.

**Fig. 1:**
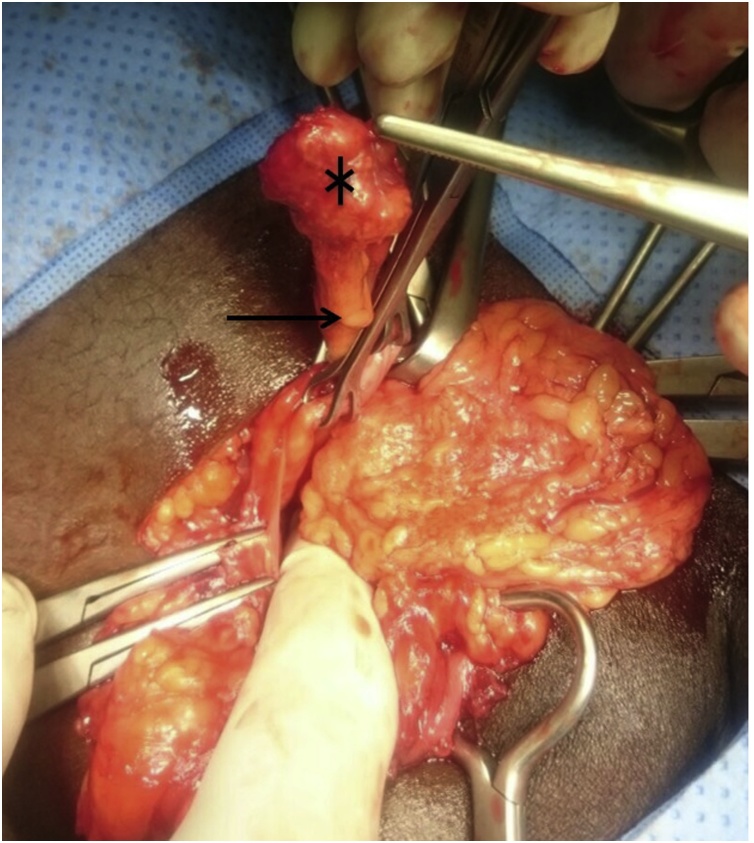
Appendix (arrow) inside hernia sac with mass on the tip (*).

**Fig. 2:**
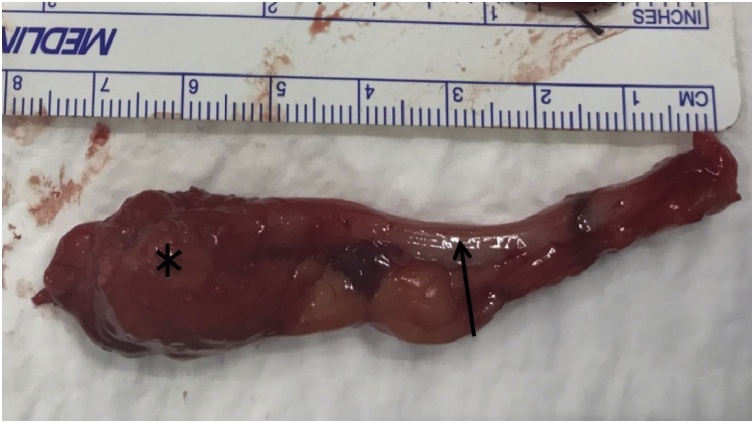
Appendix (arrow) with mass (*) after appendectomy.

The patient had normal vital signs. Abdominal examination showed a right groin swelling, not reaching down the scrotum, with no skin changes or discoloration, exhibiting a cough impulse but incompletely reducible, yet not tender. Genitourinary examination was otherwise unremarkable. These findings were clinically in favor of inguinal hernia. Laboratory tests were not done as the patient was young with no comorbidities. He was booked for daycare right inguinal mesh hernioplasty (Lichtenstein repair) [10]. The patient was prepared in the classic manner for the surgery under general anesthesia. After incision and dissection, the hernia sac was found to be thick. Palpation revealed a texture of hard mass inside. The sac was opened distally, revealed the appendix with a hard mass on its tip (Fig. 1, Fig. 2), and the cecum formed part of the sac wall proximally. Appendectomy was carried out from the same incision, followed by reduction of the cecum to the peritoneal cavity and closure of the sac, then completion of the hernia repair. Post-operative diagnosis was right indirect sliding Amyand Hernia with mass. The patient recovered smoothly and was discharged. Follow up was arranged 10 days after discharge in order to review the histopathology report.

**Fig. 3:**
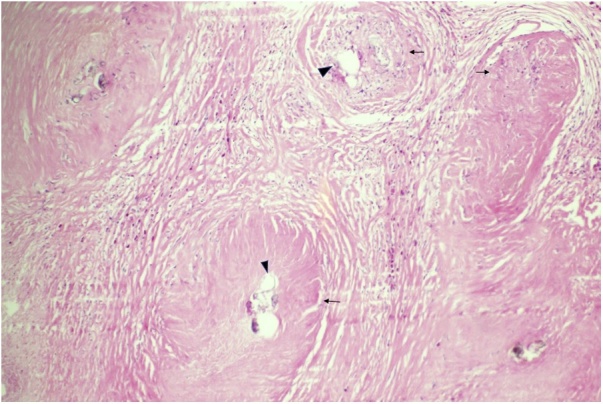
Medium-power view of periappendiceal tissue showing fibrosis and granulomas (arrows) surrounding calcified schistosoma ova (arrowheads) (H&E stain).

**Fig. 4:**
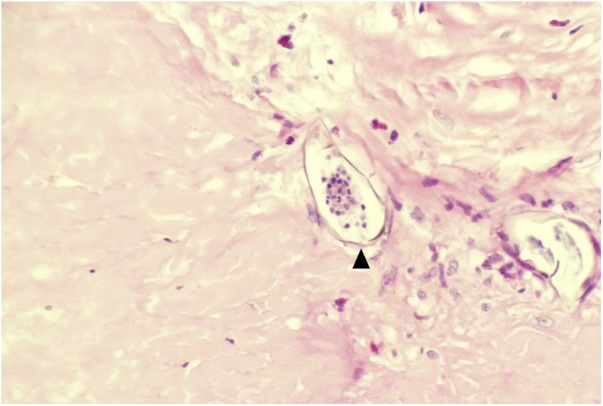
High-power view of a calcified schistosoma ovum surrounded by fibrosis and scanty inflammatory cells (H&E stain).

At follow up, the wound was healing well with no surgical complications, histopathology showed periappendiceal necrotizing granulomatous inflammation with calcified parasitic ova, consistent with schistosomiasis (Fig. 3, Fig. 4). Further subtyping of the Schistosoma was not possible due to the calcification of ova. The patient was informed about the histopathologic findings and was referred to infectious disease clinic.

## 3 Discussion

Amyand’s hernia (AH) is rare [4], schistosomiasis of the appendix is very uncommon [7], and both conditions coexisting together is an extremely rare event. To the best of our knowledge, the current paper is first to report both these two conditions in coexistence.

In terms of presentation, AH usually presents as usual inguinal hernia with no specific or characteristic clinical features; the same applies to the presentation of the appendix inside the AH, unless the appendix is inflamed [4]. Hence the diagnosis of such combination requires the physician to have a rather high index of suspicion [4]. Our case presentation is in agreement with published cases of AH in terms of groin swelling indicating inguinal hernia that requires surgical repair [11,12].

As for investigations, preoperative evaluation may be uncertain in cases where there is concomitant pathology on top of AH [13]. Ultrasound and computed tomography (CT) may assist the diagnosis of AH and subsequent management. However, these investigations are not routinely undertaken for most hernia cases unless there is suspicion of something else or the diagnosis is not clinically definite [3]. In line with such strategy, our case was booked for surgery without imaging investigations. AH is usually an intraoperative finding [4,11,12], and indeed our diagnosis of AH was intraoperative.

Intraoperatively, the initial exploration and palpation showed that the indirect sac was thick and with a hard small mass within it. Hence, we decided to open the sac distally instead of proximally in order to avoid injury to any sliding organ should there be one. In our case, the thick hernia sac was the wall of the cecum. Such approach is in agreement with the treatment of sliding hernias [14]. Intraoperatively, four categories of AH exist, based on the status of the appendix in the hernia [15]. Type 1, normal appendix in inguinal hernia; Type 2, acute appendicitis in inguinal hernia, no abdominal sepsis; Type 3, acute appendicitis with peritonitis; and, Type 4, Acute appendicitis in inguinal hernia with abdominal concomitant pathology [15]. Intraoperatively, our case was Type 1, where gross appearance showed a non-inflamed appendix. The small hard mass on the tip of the appendix raised the suspicion of neoplastic or infectious (e.g. Schistosomiasis) etiology, as the patient was from a schistosoma-endemic area.

As for the surgical procedure, Losanoff and Basson proposed the surgical treatment of AH depending on its type [15]. These include: hernia reduction and mesh repair without appendectomy to avoid risk of mesh infection (Type 1); appendectomy and primary repair of the hernia without mesh (Type 2); laparotomy and appendectomy and hernia repair without mesh (Type 3); and, Type 4 is as type 3 plus management of concomitant disease [15]. Many reports support that Type 1 does not require appendectomy because of the risk of infectious complications [3,15], while some authors favor performing appendectomy even for Type 1 with normal appendix [12,16]. Our case was Type 1 AH but with a mass on the tip which is not previously described in literature. As our deferential included both neoplastic and infective etiologies, so appendectomy was carried out. The mass involved only the tip, and the remaining appendix and cecum looked healthy, so no bowel parts were resected apart from the appendix and its mesentery for diagnostic and therapeutic purposes.

Postoperatively, histopathology of the resected specimen revealed AS, which was an incidental finding. The patient was an emigrant from an endemic schistosomiasis area, in agreement with other reported AS [17,18]. Our patient had no symptoms suggestive of AS (e.g. abdominal pain, gastrointestinal or urinary symptoms) unless his inguinal discomfort is considered to be due to the chronic inflammation caused by Schistosoma and not attributable mainly to the hernia. Absence of abdominal pain is not common in AS, as ≈80% of AS present with the clinical picture of acute appendicitis or chronic right iliac fossa pain [19]. Most AS patients do not have urinary symptoms [18], and blood investigations are rarely conclusive, sometimes showing only eosinophilia [18]. In agreement with others, definitive schistosomiasis diagnosis of our case was based on the post-operative histopathologic findings [8].

The treatment of AS is appendectomy and a single dose of praziquantel [7]. After the histopathology confirmation of schistosomiasis, we referred the patient to the infectious disease clinic. Although serious complications have been described, the prognosis of AH is generally good [3]. Our surgical choice in this case led to uneventful outcome. A longer follow up period would have revealed if any infection would have developed in such inguinal mesh hernioplasty that we undertook.

## 4 Conclusions

Diagnosis of AH requires a high index of suspicion as the history may not be typical either for acute appendicitis or incarcerated hernia, and physical examination maybe inconclusive. Intraoperative identification of non-typical thick hernia sac before its opening should alert the surgeon of the possibility of sliding hernia with an organ as a part of the sac. Rare causes of appendicular tumors like schistosomiasis should be considered in endemic areas or immigrants coming from these areas, despite the difficulty of preoperative diagnosis. Management follows the general guidelines of appendectomy and hernia repair whilst dealing with any associated pathology where present. Early diagnosis and proper surgical intervention result in excellent outcomes.

## Funding

Nothing to declare.

## Ethical approval

Approved by medical research center, Hamad Medical Corporation reference number (MRC-04-19-210).

## Consent

Written informed consent was obtained from the patient for publication of this case report and accompanying images. A copy of the written consent is available for review by the Editor-in-Chief of this journal on request.

## Author’s contribution

Ali Toffaha: study concept, data collection, interpretation, writing the paper. Walid El Ansari: data interpretation, writing the paper. Orwa Elaiwy: data interpretation, writing the paper. Munzir Obaid: data interpretation, writing the paper. Omer Al-Yahri: data interpretation, writing the paper. S. Abdelazim: data interpretation, writing the paper.

## Registration of research studies

1. Name of the registry: researchregistry

2. Unique Identifying number or registration ID: researchregistry5043

3. Hyperlink to the registration: https://www.researchregistry.com/register-now#home/registrationdetails/5d3c224113e2dd0010e38582/.

## Guarantor

Ali Toiffaha: Atoffaha2@gmail.com.

Walid El Ansari: welansari9@gmail.com.

## Provenance and peer review

Not commissioned, externally peer-reviewed.

## Declaration of Competing Interest

Nothing to declare.
